# Immunocytochemical assessment of bone marrow aspirates for monitoring response to chemotherapy in small-cell lung cancer patients

**DOI:** 10.1038/sj.bjc.6690831

**Published:** 1999-12

**Authors:** G Pelosi, F Pasini, C Ottensmeier, F Pavanel, E Bresaola, A Bonetti, F Fraggetta, A Terzi, A Iannucci, G L Cetto

**Affiliations:** Department of Pathology and Laboratory Medicine, European Institute of Oncology, Via G Ripamonti 435, I-20141 Milan, Italy; Departments of Medical Oncology andPathology, University of Verona, Verona, Italy; CRC Wessex Oncology Unit, Southampton University Hospitals, NHS Trust, Southampton, UK; Departments of Pathology andThoracic Surgery, Ospedale Civile Maggiore, Verona, Italy

**Keywords:** bone marrow, immunocytochemistry, micrometastases, SCLC, chemotherapy, prognosis

## Abstract

Recent reports have suggested that tumour cell immunodetection in bone marrow of small-cell lung cancer patients is by far more frequent than found cytohistologically and may have clinical relevance. This study evaluates primarily the efficacy of chemotherapy as method of in vivo purging, but also the relationship of marrow involvement with survival. A total of 112 bone marrow aspirates from 30 chemo-naïve patients were stained twice using anti-NCAM antibodies, first at diagnosis and then after chemotherapy (24 patients) or at disease progression (six patients). Marrow contamination was associated with lower survival (*P* = 0.002), and was also detected in 7/17 patients conventionally staged as having limited disease. At multivariate analysis, marrow involvement was an independent factor of unfavourable prognosis (*P* = 0.033). The amount of tumour contamination, before and after chemotherapy, remained unchanged also in responders and even in the subset of patients with apparent limited disease. Following chemotherapy, bone marrow became tumour negative only in 25% of initially positive responders and in none of non-responders. Our results indicate that (i) chemotherapy is not effective in purging bone marrow even in chemo-responsive patients and (ii) a subset of patients with limited disease and negative bone marrow aspirates might have a more favourable prognosis. © 1999 Cancer Research Campaign


*British Journal of Cancer* (1999) **81**(7), 1213–1221
© 1999 Cancer Research Campaign
Article no. bjoc.1999.0831

### Immunocytochemical assessment of bone marrow aspirates for monitoring response to chemotherapy in small-cell lung cancer patients

**G Pelosi[1], F Pasini[2], C Ottensmeier[4], F Pavanel[2], E Bresaola[3], A Bonetti[2], F Fraggetta[1], A Terzi[6], A Iannucci[5] and GL Cetto[2]**

[1]Department of Pathology and Laboratory Medicine, European Institute of Oncology, Via G Ripamonti 435, I-20141 Milan, Italy; Departments of [2]Medical Oncology and [3]Pathology, University of Verona, Verona, Italy; [4]CRC Wessex Oncology Unit, Southampton University Hospitals, NHS Trust, Southampton, UK; Departments of [5]Pathology and [6]Thoracic Surgery, Ospedale Civile Maggiore, Verona, Italy

**Summary** Recent reports have suggested that tumour cell immunodetection in bone marrow of small-cell lung cancer patients is by far more frequent than found cytohistologically and may have clinical relevance. This study evaluates primarily the efficacy of chemotherapy as method of in vivo purging, but also the relationship of marrow involvement with survival. A total of 112 bone marrow aspirates from 30 chemo-naïve patients were stained twice using anti-NCAM antibodies, first at diagnosis and then after chemotherapy (24 patients) or at disease progression (six patients). Marrow contamination was associated with lower survival ($P$ = 0.002), and was also detected in 7/17 patients conventionally staged as having limited disease. At multivariate analysis, marrow involvement was an independent factor of unfavourable prognosis ($P$ = 0.033). The amount of tumour contamination, before and after chemotherapy, remained unchanged also in responders and even in the subset of patients with apparent limited disease. Following chemotherapy, bone marrow became tumour negative only in 25% of initially positive responders and in none of non-responders. Our results indicate that (i) chemotherapy is not effective in purging bone marrow even in chemo-responsive patients and (ii) a subset of patients with limited disease and negative bone marrow aspirates might have a more favourable prognosis. © 1999 Cancer Research Campaign

**Keywords:** bone marrow; immunocytochemistry; micrometastases; SCLC; chemotherapy; prognosis

Small-cell lung cancer (SCLC) is a highly aggressive malignancy and most patients have widespread disease at the time of diagnosis. Though the disease is usually chemo- and radiosensitive initially, responses to treatment are normally not lasting even in cases of limited disease. Moreover, since autologous bone marrow (BM) or peripheral blood progenitor cell transplantation of SCLC patients may represent an additional therapeutic option, sensitive and reproducible methods for detecting even minimal contamination levels by tumour cells are needed. A growing body of evidence suggests that not only BM contamination by SCLC cells is far more frequent than previously shown by traditional cytohistological methods (Stahel et al, 1985; Leonard et al, 1990; Pasini et al, 1994*b*, 1995) but also that BM involvement may have clinical relevance (Leonard et al, 1990; Bucher et al, 1994; Pasini et al, 1994*b*, 1995). Immunocytochemistry is a convenient method for detecting BM micrometastases thanks to its simplicity, reliability and relative low cost (Leonard et al, 1990; Molino et al, 1991; Beiske et al, 1992; Skov et al, 1992; Pasini et al, 1994*a*), whereas other techniques, such as in vitro cultures of marrow cells (Pollard et al, 1981; Hunter et al, 1987; Everard et al, 1990) or magnetic resonance imaging (Trillet et al, 1989), are cumbersome, time-consuming and expensive.

*Received 30 June 1998*
*Revised 26 April 1999*
*Accepted 29 April 1999*

*Correspondence to:* G Pelosi

Among the SCLC-related antigens, the neural cell adhesion molecule (NCAM, CD56, NKH-1) is recognized by the cluster-1 monoclonal antibodies (Hirsch and Hansen, 1980; Kelly et al, 1984; Stahel et al, 1994). NCAM belongs to a family of membrane-bound, homophilic calcium-dependent glycoproteins involved in both cell–cell and cell–substrate interactions (Keilhauer et al, 1985; Cunningham et al, 1987; Rutishauer et al, 1988; Acheson et al, 1991). They are also expressed in a wide variety of normal tissues, mainly of neural and mesenchymal lineage (Cunningham et al, 1987; Dalseg et al, 1989; Patel et al, 1991; Lanier et al, 1992), as well as in most neuroectodermal and some mesodermal tumours (Patel et al, 1989, 1991; Moolenaar et al, 1990; Tome et al, 1991; Kern et al, 1992; Miettinen and Cupo, 1993; Ledermann et al, 1994; Pasini et al, 1994*a*, 1995; Nakamura et al, 1995). Several isoforms of the molecule have been described that derive from a single copy gene by alternative mRNA splicing (Cunningham et al, 1987). The expression of these isoforms is regulated, depending on developmental stage and in a tissue-specific manner. During embryogenesis, strongly sialylated isoforms of 200–250 kDa are prevalent (embryonic type), while the less sialylated isoforms of 185, 145 and 129 kDa are found in adult tissues (Pollerberg et al, 1986). The isoforms of NCAM expressed by SCLC are mostly embryonic (Moolenaar et al, 1990; Komminoth et al, 1991; Patriarca et al, 1997). Although the biological significance of these molecules in SCLC is unknown, there is some evidence that sialylated isoforms are involved in the metastatic process owing to their reduced adhesive properties (Doyle et al, 1990; Moolenaar et al, 1990, 1992; Carbone et al, 1991; Komminoth et al, 1991; Patriarca et al, 1997).

Staging procedures for primary or recurrent SCLC generally include clinical history, physical examination, chest X-ray, total body computerized tomography (CT) scan, and laboratory findings (Abrams et al, 1988; Perez et al, 1997). Moreover, most staging protocols include BM assessment through morphological screening of mono- or bilateral core biopsies, aspiration clots or smears from iliac crests (Abrams et al, 1998). Many studies have addressed the detection of SCLC cells in bone marrow aspirates (BMA) by immunocytochemistry (Frew et al, 1986; Berendsen et al, 1988; Hay et al, 1988; Moss et al, 1988; Trillet et al, 1989; Leonard et al, 1990; Beiske et al, 1992; Skov et al, 1992; Myklebust et al, 1993*a*, 1993*b*), but the clinical relevance of this approach is still debated. Furthermore, few reports have investigated the relationship between BM involvement and prognosis (Leonard et al, 1990; Bucher et al, 1994; Pasini et al, 1994*b*, 1995, 1998), but no study has systematically assessed the effects of treatment on marrow contamination in sequential BMA. Only two papers reported earlier systemic relapse in patients with immunocytological positive BM at re-staging after chemotherapy (Hay et al, 1988) or the disappearance of bone marrow contamination after induction chemotherapy (Menard et al, 1988).

In this study we used immunocytochemistry to (i) assess marrow involvement in 30 chemo-naïve patients with SCLC in two sequential BMA, one taken at diagnosis and the other either after chemotherapy or during disease progression; (ii) evaluate the efficacy of chemotherapy in BM purging; (iii) correlate these findings with clinicopathological features. Our findings suggest that the presence and the extent of BM involvement parallel the clinical aggressiveness of the disease, and that chemotherapy seems ineffective in purging BM.

### MATERIALS AND METHODS

### Patients, staging and treatment

From our series of BMA from 119 chemo-naïve SCLC patients collected at the Divisione Clinicizzata di Oncologia Medica of the University of Verona between March 1990 and May 1997 we chose 30 patients. The selection criteria included: (i) the availability of enough unstained cytospin obtained from two consecutive BMA in the same patient, one at the time of diagnosis and the other at restaging; and (ii) the presence in each cytospin of a high cellularity composed of well-preserved and monolayered mononuclear cells. In fact, only the use of high-quality cytospins containing at least $1 \times 10^5$ mononuclear cells per patient allowed quantitative estimation of tumour cells to be performed with reasonable confidence.

The clinical and pathological data of the 30 patients are summarized in Table 1. The diagnosis of SCLC was made on transbronchial biopsies (22 cases), fine needle aspiration biopsies (three cases), surgically excised lungs (three cases) and lymph node biopsies (two cases). All patients were staged by evaluating clinical history, physical examination, chest X-ray, total body CT scan, haemogram, routine laboratory profile and bilateral BM biopsies. BM aspirations were performed after marrow biopsy, stored and not evaluated for staging purposes. At diagnosis patients were classified as having limited disease (LD, 17 cases) if tumour was restricted to one hemithorax with regional metastases to hilar, ipsilateral or contralateral mediastinal, supraclavicular lymph nodes and/or with ipsilateral pleural effusion. By definition, BM biopsies in these cases were negative. Extensive disease (ED, 13 cases) was diagnosed if tumour extended beyond these sites. At restaging, disease status was assessed according to standard criteria as follows: complete remission (CR), partial remission (PR), stable disease (SD) and progressive disease (PD). Near complete remission (NCR) was defined as partial remission greater than 90%.

The first BMA was always obtained at diagnosis before starting chemotherapy. The second sample was taken in 24 patients after completion or at least after three cycles of chemotherapy and in six at disease progression far from chemotherapy. Nineteen patients were treated with CAVE (cyclophosphamide, doxorubicin, vincristine and etoposide), six with HD-ICE (high-dose ifosfamide, carboplatinum and etoposide), three with cisplatinum and etoposide, and two with single-agent etoposide or teniposide. In the analysis of the effects of chemotherapy, we have grouped together CR, NCR and PR as the responder group, and SD and PD as the non-responder group. The six patients assessed later at disease progression were not considered for response to treatment.

### Immunocytochemical procedures

Diagnostic material consisted of 112 BMA (3–5 ml each) obtained from posterior iliac crests. All patients were analysed twice, with bilateral aspirates in 26 (104 samples) and unilateral in four (eight samples). These samples were sedimented onto a Ficoll-Hypaque (Pharmacia, Uppsala, Sweden) density gradient at 400 ***g*** for 30 min at 19°C. The mononuclear cell layer was washed three times in RPMI-1640 (MA Bioproducts, Walkersville, MD, USA) supplemented with 15% heat-inactivated fetal calf serum (FCS; 350 ***g*** for 7 min at 4°C), and then resuspended at $1–1.5 \times 10^6$ cells $ml^{-1}$. One hundred microlitres of this cell suspension were cytospun (Cytospin 3®, Shandon Scientific Limited, Cheshire, England) using 500 rpm for 4 min at room temperature. This procedure gave a monolayered spot of 0.6 cm in diameter, composed of $50–75 \times 10^3$ well-preserved mononuclear cells for each slide. The slides were fixed in cold acetone for 5 min.

The tumour cells were characterized by the expression of NCAM using two antibodies, clone NCC-LU243 ($IgG_{2a}$) and NCC-LU-246 ($IgG_1$) (Nippon Kayaku Co, Tokyo, Japan). The specificity of the two antibodies was reported with adjacent epitopes of the 145- and 185-kDa-human isoforms of NCAM (Hirano et al, 1989; Moolenaar et al, 1990), as well as of the highly sialylated isoforms (Moolenaar et al, 1990). Immunostaining procedure was derived from Davidoff's 'sandwich' method (Davidoff et al, 1991) with minor modifications. Briefly, after primary antibody incubation (20 μl $ml^{-1}$ for 1 h), biotinylated horse anti-mouse antibody (3 μg $ml^{-1}$ for 30 min; Vector Laboratories, Burlingame, CA, USA), alkaline phosphatase–anti-alkaline phosphatase mouse complex (20 μg $ml^{-1}$ for 30 min; Dakopatts, Glostrup, Denmark), again biotinylated horse anti-mouse antibody, and streptavidin alkaline phosphatase complex (3 μg/ml for 30 min; Southern Biotechnology Associates, Birmingham, AL) were added in sequence. Blocking of endogenous alkaline phosphatase activity and development of alkaline phosphatase activity of immunocomplexes were performed as previously described in detail (Pelosi et al, 1997). Slides were lightly counterstained with 1% Harris haematoxylin. The specificity of all reactions was verified by replacing primary antibodies with unrelated mouse IgG in buffer at a comparable dilution.

 © 1999 Cancer Research Campaign

**Table 1** Clinicopathological data

| Patient no. | Age/sex | Diagnosis[a] | Stage[b] | BMA[c] 1st | BMA[c] 2nd | Metastasis localization[d] Li | AG | B | BMB | LN | CNS | Relation to treatment[e] | Disease status[f] | Survival (months) |
|---|---|---|---|---|---|---|---|---|---|---|---|---|---|---|
| 1 | 61/M | TBB | LD | – | – | – | – | – | – | – | – | ACT | CR | 53+ |
| 2 | 53/M | LuFNAC | LD | – | + | – | – | – | – | – | – | ACT | CR | 15 |
| 3 | 56/M | Lu | LD | + | + | – | – | – | – | – | – | ACT | CR | 15 |
| 4 | 47/F | Lu | LD | – | – | – | – | – | – | – | – | ACT | CR | 54+ |
| 5 | 65/M | TBB | LD | – | – | – | – | – | – | – | – | ACT | CR | 42+ |
| 6 | 58/M | TBB | LD | + | + | – | – | – | – | – | – | ACT | NCR | 19 |
| 7 | 48/F | TBB | LD | – | – | – | – | – | – | – | – | ACT | NCR | 16 |
| 8 | 60/M | TBB | LD | – | – | – | – | – | – | – | – | ACT | NCR | 16 |
| 9 | 60/M | TBB | LD | – | – | – | – | – | – | – | – | ACT | PR | 17 |
| 10 | 74/M | TBB | ED | + | + | – | – | – | + | +(M) | – | ACT | PR | 11 |
| 11 | 57/M | TBB | ED | + | – | + | – | + | + | – | – | ACT | PR | 9 |
| 12 | 61/M | TBB | LD | + | + | – | – | – | – | – | – | ACT | PR | 18 |
| 13 | 67/M | TBB | ED | + | + | – | – | + | + | +(M) | – | ACT | SD | 9 |
| 14 | 58/M | TBB | ED | + | + | – | – | – | – | +(S) | – | ACT | SD | 9+ |
| 15 | 62/M | Lu | LD | – | + | – | – | – | – | – | – | ACT | PD | 5+ |
| 16 | 59/M | TBB | ED | + | + | + | – | – | + | +(M) | – | ACT | PD | 5 |
| 17 | 65/M | TBB | LD | + | + | – | – | – | – | – | – | ACT | PD | 11 |
| 18 | 66/F | LNB | ED | + | – | – | – | – | – | +(MA) | – | DCT | PR | 9 |
| 19 | 58/M | TBB | ED | + | + | + | – | – | + | – | – | DCT | PR | 6 |
| 20 | 55/M | LuFNAC | ED | + | + | – | + | – | – | +(M) | + | DCT | SD | 9 |
| 21 | 61/M | LiFNAC | ED | + | + | + | – | + | + | – | – | DCT | SD | 9 |
| 22 | 66/F | TBB | ED | + | + | + | – | – | – | – | + | DCT | PD | 4 |
| 23 | 42/F | TBB | ED | – | + | – | – | – | – | +(S) | – | DCT | PD | 5 |
| 24 | 62/M | LNB | ED | + | + | + | – | – | + | +(A) | – | DCT | PD | 9 |
| 25 | 54/M | TBB | LD | + | + | – | – | – | – | – | – | FFCT | PD | 10 |
| 26 | 59/M | TBB | LD | + | + | – | – | – | – | – | – | FFCT | PD | 17 |
| 27 | 48/M | TBB | LD | – | + | – | – | – | – | – | – | FFCT | PD | 30 |
| 28 | 42/M | TBB | ED | + | + | – | – | + | – | +(M) | + | FFCT | PD | 10 |
| 29 | 50/M | TBB | LD | + | + | – | – | – | – | – | – | FFCT | PD | 9 |
| 30 | 68/M | TBB | LD | – | + | – | – | – | – | – | – | FFCT | PD | 10+ |

[a]TBB, trans-bronchial biopsy; LNB, lymph node biopsy; LuFNAC, lung fine needle aspiration cytology; Lu, surgically excised lung; LiFNAC, liver fine needle aspiration cytology. [b]Stage of disease at diagnosis: LD, limited disease; ED, extensive disease. [c]BMA, reactivity of bone marrow aspirates for tumour cells: –, negative; +, positive. [d]Metastasis distribution at diagnosis: –, absent; +, present; Li, liver; AG, adrenal gland; B, skeletal bone; BMB, bone marrow biopsy; LN, lymph nodes (S=supraclavicular; M=mediastinal; MA=mediastinal and abdominal; A=abdominal); CNS, central nervous system. [e]Relationship between the 2nd BMA and the treatment: ACT, after chemotherapy; DCT, during chemotherapy; FFCT, far from chemotherpy. [f]Disease status was evaluated on the occasion of the second BMA. CR, complete remission; NCR, near complete remission; PR, partial remission; SD, stable disease; PD, progressive disease.

Cytospins of N592 cell line stained in parallel were employed as external positive control, whereas mononuclear cells of each BMA were used as internal negative control. Moreover, BMA from healthy volunteers and patients with unrelated, non-malignant diseases provided the control group for non-carcinoma patients. In a set of experiments we used BMA from healthy volunteers and from patients with unrelated, non-malignant diseases artificially contaminated with a known number of tumour cells at different dilutions in order to assess the sensitivity of our immunocytochemical method in the detection of NCAM-reactive tumour cells as described elsewhere (Pelosi et al, 1999). Results of these artificial contamination tests showed that NCAM-positive tumour cells were detected in expected quantities in all mixtures up to the final dilution of $1/10^5$ bone marrow cells (data not shown). Double immunostains for NCAM and low molecular weight cytokeratins were carried out in both BMA and artificial mixtures to differentiate, in doubtful cases, true tumour epithelial cells from other NCAM-positive non-tumour cells, including natural killer (NK) cells, as described elsewhere (Pelosi et al, 1999).

### Evaluation and scoring criteria of immunostained SCLC cells

All counts of labelled cells were performed independently and blindly by two observers (GP and FP) at a magnification of ×400, without knowledge of patient identity or stage of disease. Cells reactive for NCAM were considered neoplastic if a continuous and thick ring of staining was apparent throughout the cell membrane, and the cell morphology was consistent with that of epithelial tumour cells. Although CD56 is expressed in the BM also by some lymphoid cells including NK cells, the NCAM membrane and intensity pattern is considerably different between tumour and lymphoid cells (see Results). In every patient, two to four slides were examined for each antibody and for each time point. The number of NCAM-reactive tumour cells among $10^5$ mononuclear cells was assessed by each observer for each antibody by counting every case twice: the higher score for each antibody and the higher score between the two observers were thus reported. Immunoreactivity for cytokeratins resulted in a strong labelling of the cell cytoplasm.

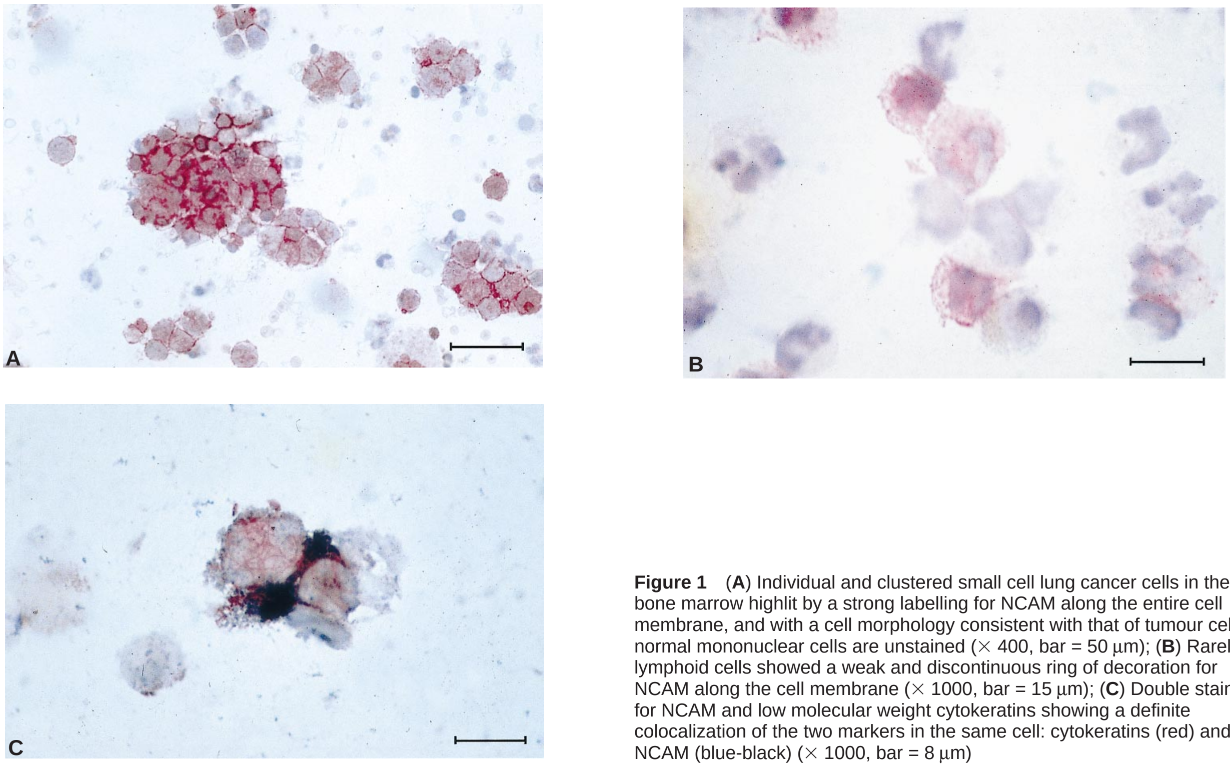

**Figure 1** (**A**) Individual and clustered small cell lung cancer cells in the bone marrow highlit by a strong labelling for NCAM along the entire cell membrane, and with a cell morphology consistent with that of tumour cells; normal mononuclear cells are unstained (× 400, bar = 50 μm); (**B**) Rarely, lymphoid cells showed a weak and discontinuous ring of decoration for NCAM along the cell membrane (× 1000, bar = 15 μm); (**C**) Double staining for NCAM and low molecular weight cytokeratins showing a definite colocalization of the two markers in the same cell: cytokeratins (red) and NCAM (blue-black) (× 1000, bar = 8 μm)

### Statistical analysis

The statistical tests used were Mann–Whitney's test and Fisher's exact *t*-test. The intra- and interobserver reproducibility was evaluated by analysis of variance and Spearman's rank test respectively. Survival curves were calculated with Kaplan–Meier's method (Kaplan and Meier, 1958) and compared by the log-rank test (Mantel and Haenszel, 1959). Multivariate analysis was carried out by means of the proportional hazards Cox-model (Cox, 1972). The following variables were examined: stage of disease (patients with LD and negative BMA vs patients with LD and positive BMA vs patients with ED); BMA contamination after chemotherapy (negative vs positive); disease status (responders vs non-responders); age; and sex. In all tests, *P*-value was considered as significant at the ≤ 0.05 level. All significance levels were of the two-sided type.

### RESULTS

Tumour cells in bone marrow were highlit by a strong membrane labelling and could be seen not only as individual cells but also as aggregates of various sizes. Both individual and clustered tumour cells were generally larger than normal mononuclear cells. Control experiments showed that normal bone marrow elements on cytospin preparations of both clinical material and control group of non-cancer patients were completely negative for NCAM, although a few lymphoid cells showed a weak and discontinuous ring of staining along the cell membrane. Results of double immunostaining for NCAM and cytokeratins confirmed a definite colocalization of the two markers in the same cells. These features are illustrated in Figure 1 A–C.

In our series, 17 patients had limited disease, and 13 extensive disease. The results of quantitative estimations of marrow contamination, clinical and pathological correlations, and survival analysis are summarized in Tables 2–7.

There was no significant difference in the number of NCAM-reactive tumour cells between the two antibodies, and the cases of positive and negative BMA were identical for both antibodies. Moreover, no differences were found in the distribution of tumour cells between the two aspiration sites. Overall, BMA were positive in 19/30 (63%) patients at onset and in 22/30 (73%) at re-staging. The order of magnitude of BM involvement at diagnosis and re-staging is reported in Table 2. The wide range of tumour cells was due to two patients (no. 19 and no. 29) who showed a high level of bone marrow contamination at diagnosis and at progression respectively. Intraobserver reproducibility did not show statistically significant differences in the estimated number of tumour cells, and a high grade of correlation between the two observers was found ($P < 0.001$, 95% confidence interval (CI) 98–100%).

Overall positivity of BMA at diagnosis was significantly associated only with extensive disease ($P = 0.005$), liver metastases ($P = 0.045$) and positive conventional BM biopsy ($P = 0.025$) (Table 3).

At diagnosis, the 17 patients with LD showed a mean level of BM involvement lower than that found in the 13 patients with ED (116 vs 1590 cells, $P = 0.008$) (Table 4). However, in the LD patients there was a prevalence of negative marrow specimens (58.8%) compared to ED patients (7.7%). The level of contamination in the LD patients with positive BMA, though apparently lower, did not differ statistically from that of ED patients (281 vs 1590 cells respectively). The difference remained not significant even after exclusion of responsive patient who showed the highest level of bone marrow contamination at diagnosis (not shown).

At the time of re-staging, marrow infiltration in LD patients, compared with that of the first aspirate, remained unchanged in ten patients showing remission of disease (53 vs 62 cells), and

 © 1999 Cancer Research Campaign

**Table 2** NCC-LU-243 and NCC-LU-246 reactivities as a function of the time point of BMA

| | | NCC-LU-246 | | NCC-LU-243 | |
|---|---|---|---|---|---|
| | | Tumour cells[a] | | Tumour cells[a] | |
| **Time point** | **Positive cases** | **Mean ± s.d.** | **Range** | **Mean ± s.d.** | **Range** |
| At diagnosis (1st BMA) | 19/30 | 1061 ± 2230 | 10–9418 | 1175 ± 2276 | 15–9750 |
| At re-staging (2nd BMA) | 22/30 | 1739 ± 4499 | 10–20 000 | 1873 ± 4389 | 30–20 000 |

[a]Number of NCAM-reactive tumour cells among 100 000 mononuclear cells.

**Table 3** Characteristics of the patients in relation to BM involvement

| **Variable** | **Category** | **BMA** | **Cases** | **% BMA-positive** | ***P*-value** |
|---|---|---|---|---|---|
| Stage | LD | 1st | 17 | 41.2 | |
| | ED | 1st | 13 | 92.3 | 0.005 |
| Liver metastases | Present | 1st | 6 | 100 | |
| | Absent | 1st | 24 | 54.2 | 0.045 |
| BM biopsy | Positive | 1st | 7 | 100 | |
| | Negative | 1st | 23 | 52.2 | 0.025 |
| Disease status | Responders[a] | 2nd | 14 | 42.8 | |
| | Non-responders[a] | 2nd | 10 | 100 | 0.004 |
| Sex | Male | 1st | 25 | 68 | |
| | Female | 1st | 5 | 40 | NS |
| Age (median value) | ≤59 years | 1st | 16 | 68.7 | |
| | >59 years | 1st | 14 | 57.1 | NS |

[a]Ten of the 14 responders had LD and eight of the ten non-responders had ED ($P = 0.036$).

**Table 4** Number of tumour cells in BMA as a function of stage, disease evolution and treatment

| | | | BMA at diagnosis | | BMA at re-staging | | |
|---|---|---|---|---|---|---|---|
| | | **No. of cases** | **Mean ± s.d.** | **Median** | **Mean ± s.d.** | **Median** | ***P*-value** |
| Stage | Limited disease | 17 | 116 ± 253 | 0 | – | – | 0.008 |
| | Extensive disease | 13 | 1590 ± 2675 | 204 | – | – | |
| Evolution | LD with progressive disease | 7 | 205 ± 372 | 32 | 3968 ± 7306 | 363 | 0.026 |
| | LD with disease remission | 10 | 53 ± 104 | 0 | 62 ± 94 | 0 | NS |
| | ED with progressive disease | 9 | 1195 ± 1459 | 58 | 1300 ± 2091 | 167 | NS |
| | ED with partial response | 4 | 2477 ± 4628 | 238 | 277 ± 450 | 82 | NS |
| Treatment | Responders | 14 | 746 ± 2498 | 8 | 124 ± 251[a] | 0 | NS |
| | Non-responders | 10 | 909 ± 1444 | 45 | 1229 ± 1982[a] | 220 | NS |

[a]$P = 0.006$.

**Table 5** Results of BMA in relation to stage and time point of evaluation

| | | | | At diagnosis | At re-staging |
|---|---|---|---|---|---|
| **Patients** | | **Stage** | **No.** | **+/–** | **+/–** |
| All patients | | LD | 17 | 7/10 | 11/6 |
| | | ED | 13 | 12/1 | 11/2 |
| | Total | | 30 | 19/11 | 22/8 |
| Responders | | LD | 10 | 3/7 | 4/6 |
| | | ED | 4 | 4/0 | 2/2 |
| | Total | | 14 | 7/7 | 6/8 |
| Non-responders | | LD | 2 | 1/1 | 2/0 |
| | | ED | 8 | 7/1 | 8/0 |
| | Total | | 10 | 8/2 | 10/0 |

increased in seven with progressive disease (205 vs 3968 cells, $P = 0.026$). In ED patients, there were no significant differences between the two aspirates, though the number of tumour cells was lower in the four patients achieving partial response (Table 4). This cohort of patients, however, is too small to be analysed separately.

The relationship between treatment and BMA involvement was assessed for the 24 patients (from patient 1 to patient 24, Table 1), who were restaged after completion or after at least three cycles of chemotherapy. Of these, 14 were responders (five CR, three NCR, six PR) and ten non-responders (four SD and six PD) (Table 5).

In the group of the 14 responders, the number of contaminating cells did not significantly change between the first and the second aspirate, though there was a reduction of the mean marrow contamination (746 vs 124 cells) (Table 4). The difference remained not

© 1999 Cancer Research Campaign 

**Table 6** Results of actuarial survival analysis in 30 patients

| Variable | Category | Median survival | *P*-value |
|---|---|---|---|
| 1st BMA | Negative | 16.5 | |
| | Positive | 9 | 0.002 |
| Stage | LD | 16.5 | |
| | ED | 9 | <0.001 |
| 2nd BMA | Negative | 16 | |
| | Positive | 10 | 0.023 |
| Treatment | Responders | 16 | |
| | Non-responders | 9 | 0.027 |

significant even excluding the responsive patient who showed the highest level of contamination at diagnosis (not shown). Ten of these patients at diagnosis had LD, with seven of them showing also a negative BMA ($P = 0.036$) (Tables 3 and 5). Among responders, the second aspirate became positive in one of the seven negative LD patients and negative in two of the four ED patients, while in one patient there was an increase in the number of contaminating cells (Tables 1 and 5 and Figure 2). Even in the non-responder patients, the level of contamination was not different between the first and the second aspirate (909 vs 1229 cells) and was also higher than in responders (1229 vs 124, $P = 0.006$) (Table 4). All but two of the non-responders had ED and all became positive at the time of the second aspirate ($P = 0.036$) (Tables 3 and 5), though two patients showed a certain reduction of the degree of contamination (Figure 2).

Regarding survival, only positive BMA at diagnosis, extensive disease, positive BMA at re-staging, and lack of response significantly affected survival (Table 6). Combining stage of disease and BMA immunoreactivity at diagnosis, three groups with different prognosis could be identified: group A, with LD and negative BMA (ten patients; median survival: 17 months) ('true LD'); group B, with LD and positive BMA (seven patients; median survival: 13 months) ('untrue LD'); and group C, with ED and either positive or negative BMA (13 patients; median survival: 9 months). The likelihood of survival was significantly different between groups: A vs B, $P = 0.033$; B vs C, $P = 0.003$; A vs C, $P < 0.001$ (Figure 3). In a multivariate analysis, both ED (hazard ratio = 35.983, $P < 0.001$) and LD with positive BMA (hazard ratio = 7.122; $P = 0.033$) emerged as independent predictors of poor prognosis (Table 7).

### DISCUSSION

Our results can be summarized as follows: (i) BMA contamination by SCLC cells at diagnosis is related to the extension of disease and predicts poor survival; (ii) chemotherapy is ineffective in purging BM from tumour cells; and (iii) the occurrence of tumour cells in BMA of patients with LD identifies a subgroup of 'untrue LD' patients with reduced life expectation.

This clinicopathological study was mainly aimed at evaluating the relation between bone marrow involvement and clinical outcome: by screening $10^5$ mononuclear cells, we have found a correlation with survival and response to chemotherapy. Therefore this methodological approach seems acceptable in terms of sensitivity. The screening of a higher number of cells (i.e. $10^6$) requires immunostaining of many more cytospin slides, and would be therefore both time-consuming and expensive.

In this study, we found that BMA contamination by tumour cells at diagnosis is predictive of shorter survival ($P = 0.002$) (Table 5). This result, however, could reflect the dismal prognosis of ED patients, since in our series positive BMA were more frequent in patients with extensive than limited disease (92.3 vs 41.2% respectively) ($P = 0.005$). We confirmed also that marrow positivity was associated with liver metastases, as previously reported (Skov et al, 1992) (Table 3). This may imply that liver involvement in patients with positive BMA is more frequent than found with conventional staging procedures.

Consistently, we detected at least one order of magnitude more tumour cells in BMA of ED patients (1590 cells) than in those with conventionally assessed LD (116 cells) ($P = 0.008$) (Table 4). This figure is in agreement with previous reports pointing out that the persistence of residual marrow disease at clinical remission (Leonard et al, 1990), as well as the presence of tumour cells in BMA at diagnosis (Bucher et al, 1994; Pasini et al, 1994*b*, 1995, 1998), are predicitive of early metastatic relapse and reduced survival rate respectively.

**Table 7** Multivariate analysis on the relationship between survival and variables tested in 30 patients

| Predictive variables | RC | SE | HR | 95% CI | *P*-value |
|---|---|---|---|---|---|
| Stage | | | | | |
| ED (Group C) | 3.583 | 1032 | 35.983 | 4.760–272.007 | <0.001 |
| LD/BMA pos (Group B) | 1.963 | 0.921 | 7.122 | 1.172–43.286 | 0.033 |
| (baseline: LD/BMA neg [Group A]) | | | | | |
| 2nd BMA | | | | | |
| Positive | –0.002 | 0.963 | 0.998 | 0.151–6.601 | NS |
| (baseline: negative) | | | | | |
| Disease status | | | | | |
| Non-responders | –0.810 | 0.659 | 0.445 | 0.122–1.618 | NS |
| (baseline: responders) | | | | | |
| Age (median value) | | | | | |
| >59 years | –0.432 | 0.508 | 0.649 | 0.240–1.757 | NS |
| (baseline: ≤59 years) | | | | | |
| Sex | | | | | |
| Female | 1230 | 0.756 | 3420 | 0.778–15.043 | NS |
| (baseline: male) | | | | | |

RC: regression coefficient; SE: standard error; HR: hazard ratio; 95% CI: confidence intervals.

 *© 1999 Cancer Research Campaign*

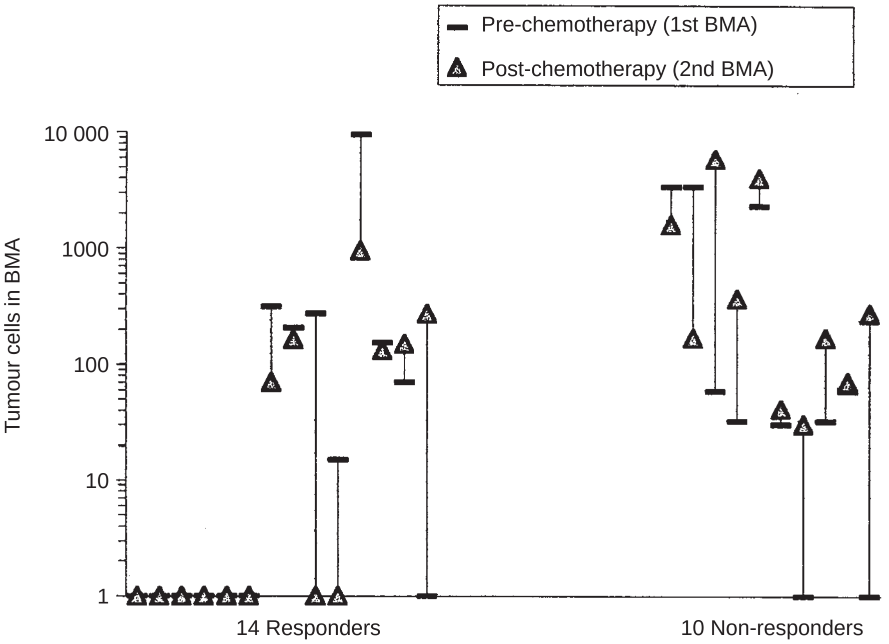

**Figure 2** Differences of NCAM-reactive tumour cells between the 1st and the 2nd BMA in 14 responders and ten non-responders to treatment. The former group had a mean decrease of 652 (s.d. = 2377) tumour cells per patient, whereas the latter group showed a mean increase of 314 (s.d. = 2267) tumour cells per patient ($P = 0.029$)

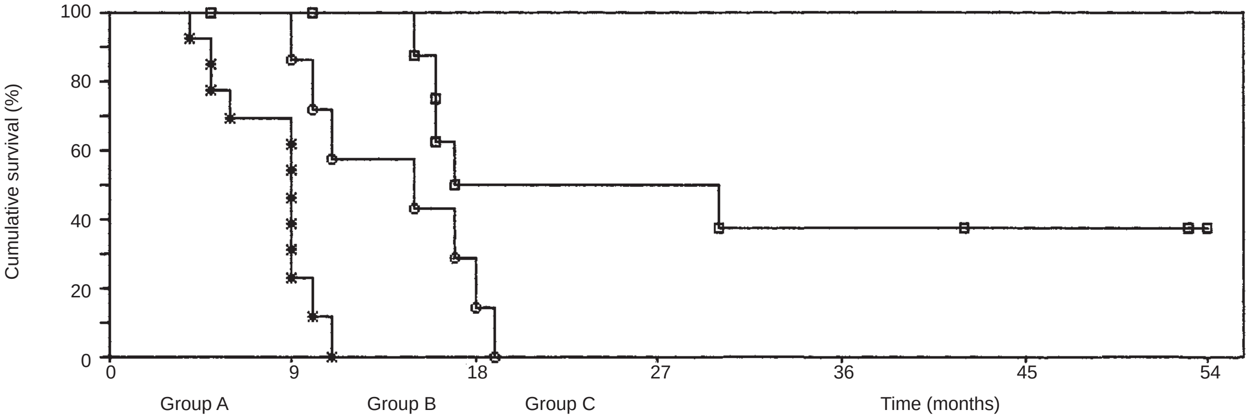

**Figure 3** Survival curves according to three different groups obtained by combining stage of disease and BMA immunoreactivity: group A, with LD and negative BMA (ten patients; median survival: 17 months) ('true LD'); group B, with LD and positive BMA (seven patients; median survival: 13 months) ('untrue LD'); and group C, with ED and either positive or negative BMA (13 patients; median survival: 9 months). Group A vs group B: $P = 0.033$; group B vs group C: $P = 0.003$; group A vs group C: $P < 0.001$

Even more challenging were the data on the relationship of tumour burden contamination of BMA with the clinical status of disease or response to chemotherapy. To the best of our knowledge, no study evaluated in sequential aspirates the effects of treatment on BM involvement. Only two reports have briefly alluded to this issue: in one, earlier systemic relapse occurred in five of eight patients with immunocytological positive BM at restaging after chemotherapy (Hay et al, 1988), and in the other, seven patients with positive pretreatment BMA became negative after induction chemotherapy (Menard et al, 1988). Moreover, we found a significant increase in the number of tumour cells in BMA of patients with conventionally assessed LD at the time of progression compared to the baseline contamination. This observation favours the hypothesis that BM involvement mirrors disease activity at many other anatomical sites (Leonard et al, 1990) rather than being a sanctuary for selective and precocious colonization by tumour cells. However, no variation was detected in non-responsive patients with ED: this group of patients was not homogeneous, because 50% of them had stable disease (Table 4). A different type of non-response to treatment (stabilization or progression) may perhaps influence the degree of BMA contamination.

In our study, five of the 11 patients (45%) originally negative became positive, mostly at the time of disease progression (four patients), reinforcing the concept that bone marrow contamination parallels the clinical course of the disease. This finding, if confirmed in a larger series, may open a new area of investigation: the

© 1999 Cancer Research Campaign detection, with repeated sampling of bone marrow, of an increase in the number of bone marrow tumour cells after chemotherapy might precociously select patients who are chemoresistant.

As one would expect, the 24 patients, in whom the second BMA was evaluable also for response to treatment, showed a reduced number of tumour cells in the bone marrow. In fact, in the second aspirate we detected a significantly lower contamination in responders than in non-responders (Table 4 and Figure 2). Among responders there was a prevalence of patients with limited disease (10/14) and negative BMA at diagnosis (7/10) (Table 5), and this is in line with the higher response rates in LD than in ED patients (Elias, 1997).

Response to treatment, however, was not associated with a complete clearance of tumour cells in BMA (Table 4). Only two of the responders became negative (both with short-lasting responses of 3 months, data not shown), two showed an increased level of contamination (one patient turning to positive), while the others remained either positive (four cases) or negative (six cases) (Figure 2). None of the non-responders became tumour-negative and all but two showed increased levels of BM contamination (Figure 2). Assuming a BM volume of 6–7% of the total body weight, it derives that we detected an initial contamination approximatively of $10^7$ tumour cells, which decreased to $10^6$ after response to treatment and increased to $10^8$ in patients with progressive disease.

It has been reported that patients with bone marrow involvement and ED have circulating tumour cells under steady-state conditions and also tumour cell recruitment upon mobilization of peripheral blood progenitor cells (Ross et al, 1993; Brugger et al, 1994). Our data do not confirm the efficacy of chemotherapy as a method for ‘in vivo’ purging the bone marrow. In the clinical setting, there is a growing consensus about the use of standard chemotherapy for both tumour debulking and bone marrow purging before high-dose chemotherapy (Brugger et al, 1994; Elias, 1997). In order to verify this hypothesis, two cooperative ongoing trials on high-dose chemotherapy in SCLC (Random-ICE, conducted by the EMBT group and the phase II trial CALGB 9331/SWOG 9433) have introduced the evaluation of contamination before and after standard chemotherapy. However, according to our results, chemotherapy is ineffective in purging bone marrow, even in responsive patients who were mostly LD. In fact a residual marrow tumour mass of about $10^6$ cells still persists after treatment. Therefore, these data support the use of further manipulation of bone marrow or peripheral blood progenitor cell products (i.e. ‘ex vivo’ purging, selection of CD34+ cells), in order to ensure a further reduction of contaminating tumour cells.

Interestingly, tumour cells were also detected in 7/17 LD patients (41.2%). This finding confirms immunocytochemistry as a more sensitive procedure in assessing BM for malignant cells in SCLC patients. In fact, also previous studies reported that 30–50% of LD patients had BMA contamination when evaluated immunocytochemically (Berendsen et al, 1988; Hay et al, 1988; Moss et al, 1988; Trillet et al, 1989; Beiske et al, 1992; Pasini et al, 1994*b*, 1995, 1998). The amount of marrow involvement of these ‘untrue LD’ patients was apparently lower than that of ED patients, though this did not achieve statistical significance. This finding is in agreement with the median survival of these patients which was intermediate between ‘true LD’ and ED patients (Figure 3). These results were also substantiated by a multivariate analysis where BMA positivity in patients with LD emerged as an independent factor of prognosis (Table 6). This is the first report documenting the existence of a subset of SCLC patients with intermediate survival substantiated by a multivariate analysis. Therefore, we suggest that the search for tumour cells in BMA by immunocytochemistry may be an easy and reproducible method for identifying subsets of patients with LD at better prognosis. These patients with ‘true LD’ (i.e. with absence of immunoreactive tumour cells in BMA) may be suitable for novel therapeutic strategies, including high-dose chemotherapy, immunotherapy and targeted immunotherapy (Myklebust et al, 1993*a*, 1993*b*). Further studies are in progress in our laboratory to confirm the role of immunocytochemistry of bone marrow among the prognostic factors already identified in large studies of SCLC patients.

### ACKNOWLEDGEMENTS

This work was supported by the Ministry for University and Scientific and Technological Research (MURST) (60%), Rome, Italy.

### REFERENCES

Abrams J, Doyle LA and Aisner J (1988) Staging, prognostic factors, and special considerations in small cell lung cancer. *Semin Oncol* **15**: 261–277

Acheson A, Sunshine JL and Rutishauer U (1991) N-CAM polysialic acid can regulate both cell–cell and cell–substrate interactions. *J Cell Biol* **114**: 143–153

Beiske K, Myklebust AT, Aamdal S, Langholm R, Jakobsen E and Fodstad Ö (1992) Detection of bone marrow metastases in small cell lung cancer patients. Comparison of immunologic and morphologic methods. *Am J Pathol* **141**: 531–538

Berendsen HH, De Leij L, Postmus PE, Ter Haar JG, Poppema S and The TH (1988) Detection of small cell lung cancer metastases in bone marrow aspirates using monoclonal antibody directed against neuroendocrine differentiation antigen. *J Clin Pathol* **41**: 273–276

Brugger W, Bross KJ, Glatt M, Weber F, Martelsmann R and Kanz L (1994) Mobilization of tumor cells and hematopoietic progenitor cells into peripheral blood of patients with solid tumors. *Blood* **83**: 636–640

Bucher M, Manegold C, Krempien B and Drings P (1994) Immunocytological staining with HEA-125 on bone marrow aspirates for staging small cell lung cancer (SCLC). *Ann Oncol* **5**: P820 (Abstract 163)

Carbone DP, Koros AMC, Linnoila I, Jewett P and Gazdar AF (1991) Neural cell adhesion molecule expression and messenger RNA splicing patterns in lung cancer cell lines are correlated with neuroendocrine phenoytype and growth morphology. *Cancer Res* **51**: 6142–6149

Cox DR (1972) Regression models and life tables. *J R Stat Soc* **34**: 187–202

Cunningham A, Hemperly JJ, Murray BA, Pridiger EA, Brackenbury R and Edelman GM (1987) Neural cell adhesion molecule: structure, immunoglobulin-like domains, cell surface modulation and alternative RNA splicing. *Science* **236**: 799–806

Dalseg AM, Linnemann D and Bock E (1989) Soluble neural cell adhesion molecule in brain, cerebrospinal fluid and plasma in the developing rat. *Int J Devl Neuroscience* **7**: 209–217

Davidoff MS, Schulze W and Holstein AF (1991) Combination of alkaline phosphatase anti-alkaline-phosphatase (APAAP)- and avidin-biotin-alkaline phosphatase complex (ABAP)-techniques for amplification of immunocytochemical staining of human testicular tissue. *Andrologia* **23**: 353–356

Doyle LA, Borges M, Hussain A, Elias A and Tomiyasu T (1990) An adherent subline of a unique small-cell lung cancer cell line downregulates antigens of the neural cell adhesion molecule. *J Clin Invest* **86**: 1848–1854

Elias AD (1997) Small cell lung cancer. State-of-the-art therapy in 1996. *Chest* **112**: 251S–258S

Everard MJ, Macaulay VM, Min T, Millar JL and Smith IE (1990) Small cell lung cancer cell line from histologically and immunocytochemically negative bone marrow. *Eur J Cancer* **26**: 766–767

 *© 1999 Cancer Research Campaign*

Frew AJ, Ralfkiaer N, Ghosh AK, Gatter KC and Mason DY (1986) Immunocytochemistry in the detection of bone marrow metastases in patients with primary lung cancer. *Br J Cancer* **53**: 555–556

Hay FG, Ford A and Leonard RCF (1988) Clinical applications of immunocytochemistry in the monitoring of the bone marrow in small cell lung cancer (SCLC). *Int J Cancer*: 8–10

Hirano T, Hirohashi S, Kunii T, Noguchi M and Shimosato Y (1989) Quantitative distribution of cluster 1 small cell lung cancer antigen in cancerous and non-cancerous tissues, cultured cells and sera. *Jpn J Cancer Res* **80**: 348–355

Hirsch FR and Hansen HH (1980) Bone marrow involvement in small cell anaplastic carcinoma of the lung. Prognostic and therapeutic aspects. *Cancer* **46**: 206–211

Hunter RF, Broadway P, Sun S, Niell HB and Mauer AM (1987) Detection of small cell lung cancer bone marrow involvement by discontinuous gradient sedimentation. *Cancer Res* **47**: 2727–2740

Kaplan EL and Meier P (1958) Nonparametric estimation from incomplete observations. *Am Stat Assoc J* **53**: 457–481

Keilhauer GA, Faissner A and Schachner M (1985) Differential inhibition of neurone–neurone, neurone–astrocyte and astrocyte–astrocyte adhesion by L1, L2 and NCAM antibodies. *Nature* **316**: 728–730

Kelly BW, Morris JF, Harwood BP and Bruya TE (1984) Methods and therapeutic value of bone marrow examination in small cell carcinoma of the lung. *Cancer* **53**: 99–102

Kern WF, Spier CM, Hanneman EH, Miller TP, Matzner M and Grogan TM (1992) Neural cell adhesion molecule-positive peripheral T-cell lymphoma: a rare variant with a propensity for unusual sites of involvement. *Blood* **79**: 2432–2437

Komminoth P, Roth J, Lackie PM, Bitter-Suermann D and Heitz PU (1991) Polysialic acid of the neural cell adhesion molecule distinguishes small cell lung carcinoma from carcinoids. *Am J Pathol* **139**: 297–304

Lanier LL, Spits H and Phillips JH (1992) The developmental relationship between NK cells and T cells. *Immunol Today* **13**: 392–395

Ledermann JA, Pasini F, Olabiran Y and Pelosi G (1994) Detection of neural cell adhesion molecule (NCAM) in serum of patients with small cell lung cancer (SCLC) with “limited” or “extensive” disease, and bone marrow infiltration. *Int J Cancer* **8**: 49–52

Leonard RCF, Duncan LW and Hay FG (1990) Immunocytological detection of residual marrow disease at clinical remission predicts metastatic relapse in small cell lung cancer. *Cancer Res* **50**: 6545–6548

Mantel N and Haenszel W (1959) Statistical aspects of the analysis of data from retrospective studies of disease. *J Natl Cancer Inst* **22**: 719–748

Menard S, Porro G, Giani S, Bedini A, Bidoli P, Santoro A, Bregni M, Gianni AM, Pilotti S, Rilke F, Soresi S, Fasolato S and Colnaghi MI (1988) Detection of SCLC metastatic cells in bone marrow by means of the monoclonal antibody MLuC1. *Lung Cancer* **4**: 73–75

Miettinen M and Cupo W (1993) Neural cell adhesion molecule distribution in soft tissue tumors. *Hum Pathol* **24**: 62–66

Molino A, Colombatti M, Bonetti F, Zardini M, Pasini F, Perini A, Pelosi G, Tridente G, Veneri D and Cetto GL (1991) A comparative analysis of three different techniques for the detection of breast cancer cells in bone marrow. *Cancer* **67**: 1033–1036

Moolenaar CECK, Muller EJ, Schol DJ, Figdor CG, Bock E, Bitter-Suermann D and Michalides RJAM (1990) Expression of neural cell adhesion molecule-related sialoglycoprotein in small cell lung cancer and neuroblastoma cell line H69 and CHP-212. *Cancer Res* **50**: 1102–1106

Moolenaar CECK, Pieneman C, Walsh FS, Mooi WJ and Michalides RJAM (1992) Alternative splicing of neural cell adhesion molecule mRNA in human small cell lung cancer cell line H69. *Int J Cancer* **51**: 238–243

Moss F, Bobrow L, Beverley P and Souhami R (1988) Detection of small cell carcinoma in bone marrow aspirates using monoclonal antibodies and mixtures of monoclonal antibodies. *Lung Cancer* **4**: 76–78

Myklebust AT, Godal A, Pharo A, Juell S and Fodstad Ø (1993*a*) Eradication of small cell lung cancer cells from human bone marrow with immunotoxins. *Cancer Res* **53**: 3784–3788

Myklebust AT, Pharo A and Fodstad Ø (1993*b*) Effective removal of SCLC from human bone marrow. Use of four monoclonal antibodies and immunomagnetic beads. *Br J Cancer* **67**: 1331–1336

Nakamura S, Suchi T, Koshikawa T, Kitoh K, Koike K, Komatsu H, Iida S, Kagami Y, Ogura M, Katoh E, Kurita S, Suzuki H, Kobashi Y, Yamabe H, Hirabayashi N, Ueda R and Takahashi T (1995) Clinicopathologic study of CD56 (NCAM)-positive angiocentric lymphoma occurring in sites other than the upper and lower respiratory tract. *Am J Surg Pathol* **19**: 284–296

Pasini F, Pelosi G, Ledermann JA and Cetto GL (1994*a*) Detection of small-cell-lung-cancer cells in bone marrow aspirates by monoclonal antibodies NCC-LU-243, NCC-LU-246 and MluC1. *Int J Cancer* 53–56

Pasini F, Pelosi G, Pavanel F, Sabbioni R, Oliani C, Mostacci R, Masotti A, Spagnoli P, Recaldin E & Cetto GL (1994*b*) Monoclonal antibodies improve the detection of bone marrow metastasis in bone marrow aspirates (BMA) of patients with small cell lung cancer (SCLC) and contribute to identify patients with better prognosis. *Ann Oncol* **5**: O808 (Abstr), 161

Pasini F, Pelosi G, Mostacci R, Santo A, Masotti A, Spagnolli P, Recaldin E and Cetto GL (1995) Detection at diagnosis of tumor cells in bone marrow aspirates of patients with small cell lung cancer and clinical correlations. *Ann Oncol* **6**: 86–88

Pasini F, Pelosi G, Verlato G, Guidi G, Pavanel F, Tummarello D, Masotti A and Cetto GL (1998) Positive immunostaining with MLuC1 of bone marrow aspirate predicts poor outcome in patients with small-cell lung cancer. *Ann Oncol* **9**: 181–185

Patel K, Frost G, Kiely F, Phimister E, Coakham HB and Kemshead JT (1991) Expression of the cluster 1 antigen (neural cell adhesion molecule) in neuroectodermal tumours. *Br J Cancer* **63**: 20–23

Patel K, Moore SE, Dickson G, Rossell RJ, Beverley PC, Kemshed JT and Walsh FS (1989) Neural cell adhesion molecule (NCAM) is the antigen recognized by monoclonal antibodies of similar specificity in small-cell lung carcinoma and neuroblastoma. *Int J Cancer* **44**: 573–578

Patriarca C, Pruneri G, Alfano MR, Carboni N, Ermellino L, Guddo F, Buffa R, Siccardi AG and Coggi G (1997) Polysialylated N-CAM, chromogranin A and B, and secretogranin II in neuroendocrine tumours of the lung. *Virchows Arch* **430**: 455–460

Pelosi G, Molino AM, Pavanel F, Turazza M and Cetto GL (1997) Immunodetection of breast cancer cells in bone marrow for monitoring high-dose sequential chemotherapy. *Appl Immunohistochem* **5**: 67–70

Pelosi G, Pasini F, Pavanel F, Bresaola E, Schiavon I and Iannucci A (1999) Effects of different immunolabeling techniques on the detection of small cell lung cancer cells in bone marrow. *J Histochem Cytochem* (in press)

Perez EA, Loprinzi CL, Sloan JA, Owens DT, Novotny PJ and Bonner JA (1997) Utility of screening procedures for detecting recurrence of disease after complete response in patients with small cell lung carcinoma. *Cancer* **80**: 676–680

Pollard EB, Tio F, Myers JW, Clark G, Coltman CA and von Hoff D (1981) Utilization of a human tumor cloning system to monitor for marrow involvement with small cell carcinoma of the lung. *Cancer Res* **41**: 1015–1020

Pollerberg GE, Schachner M and Davoust J (1986) Differentiation-state dependent surface mobilities of two forms of the neural cell adhesion molecule. *Nature* **234**: 462–465

Ross AA, Cooper BW, Lazarus HM, Mackay W, Moss TJ, Ciobanu N, Tallman MS, Kennedy MJ, Davidson NE, Sweet D, Winter C, Akard L, Jansen J, Copelman E, Meagher RC, Herzig RH, Klumpp TR, Kahn DG and Warner NE (1993) Detection and viability of tumor cells in peripheral blood stem cell collections from breast cancer patients using immunocytochemical and clonogenic assay techniques. *Blood* **82**: 2605–2610

Rutishauer U, Acheson A, Hall AK, Mann DM and Sunshine J (1988) The neural cell adhesion molecule (N-CAM) as a regulator of cell–cell interactions. *Science* **240**: 53–57

Skov BG, Hirsh FR and Bobrow L (1992) Monoclonal antibodies in the detection of bone marrow metastases in small cell lung cancer. *Br J Cancer* **65**: 593–596

Stahel RA, Gilks WR, Lehmann H and Schenker T (1994) Third international workshop on lung tumor and differentiation antigens: overview of the results of the central data analysis. *Int J Cancer* **8**: 6–26

Stahel RA, Mabry M, Skarin AT, Speak J and Bernal SD (1985) Detection of bone marrow metastasis in small cell lung cancer by monoclonal antibody. *J Clin Oncol* **3**: 455–461

Tome Y, Hirohashi S, Noguchi M, Matsuno Y, Kishi K, Uei Y and Shimosato Y (1991) Immunocytologic diagnosis of small-cell lung cancer in imprint smears. *Acta Cytol* **35**: 485–490

Trillet V, Revel D, Combaret V, Favrot M, Loire R, Tabib A, Pages J, Jacquemet P, Bonmartin A, Mornex JF, Cordier JF, Pinet F, Amiel M and Brune J (1989) Bone marrow metastases in small cell lung cancer: detection with magnetic resonance imaging and monoclonal antibodies. *Br J Cancer* **60**: 83–88

© 1999 Cancer Research Campaign 
